# Rapid growth of calcified amorphous tumor with mitral annulus calcification: a case report

**DOI:** 10.1186/s44215-024-00164-4

**Published:** 2024-08-31

**Authors:** Satoki Ozoe, Yutaka Koyama, Masahiro Inagaki, Shinji Tomita

**Affiliations:** Department of Cardiovascular Surgery, Gifu Heart Canter, 4-14-4 Yabuta minami, Gifu-shi, Gifu, Japan

**Keywords:** Calcified amorphous tumor, Mitral annular calcification, Hemodialysis, Transthoracic echocardiography, Dyspnea

## Abstract

**Background:**

Calcified amorphous tumor (CAT) of the heart is a rare, non-neoplastic cardiac mass with mitral valves and annuli being the most common sites. The presence of mitral annular calcification (MAC) is associated with an increased risk of stroke or other systemic embolisms. Here, we report a case of CAT showing rapid growth with MAC and investigate the link between the two.

**Case presentation:**

A 71-year-old man presented at our hospital with dyspnea and had been undergoing hemodialysis for 26 years for chronic glomerulonephritis. Transthoracic echocardiography (TTE) revealed moderate mitral stenosis with bulky MAC. Two months later, the patient developed progressive dyspnea, and follow-up TTE revealed a highly mobile mass (8 × 5 mm) attached to the left ventricular (LV) side of the posterior MAC. He underwent surgery because of congestive heart failure and a high risk of embolization. Surgical inspection revealed that the tumor was attached beneath the P3 segment of the mitral valve on the LV side and was removed. When removing the MAC, toothpaste-like contents drained from the encapsulated mass inside the MAC at the P3 segment, where the tumor was located. After reconstructing the posterior mitral annulus defect with a bovine pericardial patch, mitral valve replacement with a mechanical prosthesis, a maze procedure, and left appendage closure were performed. Histopathological examination revealed that the excised tumor contained fibrin and calcium deposits. The mass was diagnosed as a CAT.

**Conclusions:**

CAT may be one of the causes of stroke induced by MAC. Routine follow-up echocardiography should be recommended for patients with MAC, especially those undergoing hemodialysis.

## Background

Calcified amorphous tumors (CAT) of the heart are rare, non-neoplastic cardiac masses. Histologically, it is characterized by the presence of nodular calcium in the background of an amorphous degenerating fibrinous material [1]. The mitral valves and annuli are the most common sites of CAT. Patients with end-stage renal disease have a relatively high incidence of CAT [2]. Mitral annular calcification (MAC) is associated with an increased risk of stroke and other systemic embolisms. Here, we report a case of CAT showing rapid growth with MAC and evaluate the link between the two.

## Case presentation

A 71-year-old man with end-stage renal disease who had been on hemodialysis for 26 years for chronic glomerulonephritis was referred to our hospital for dyspnea. He had a medical history of angina and underwent percutaneous coronary intervention (PCI) of the left anterior descending artery, right nephrectomy for renal cancer, and laparoscopic cholecystectomy. He had a smoking history of 20 cigarettes per day for 45 years until the age of 68 y.

**Fig. 1:**
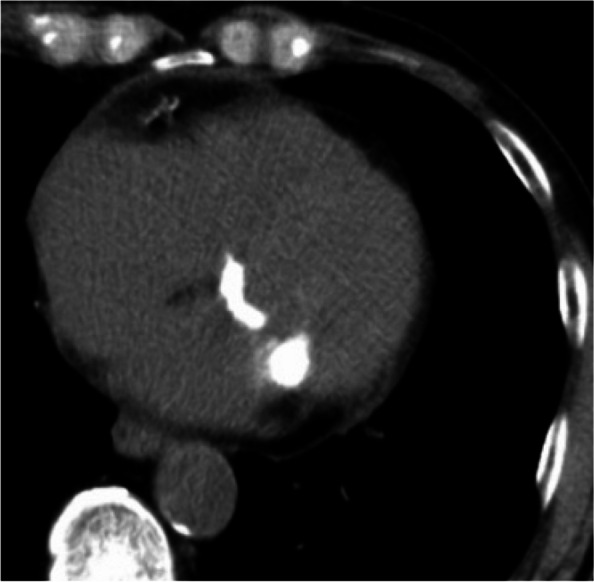
Computed tomography revealing mitral annular calcification

**Fig. 2:**
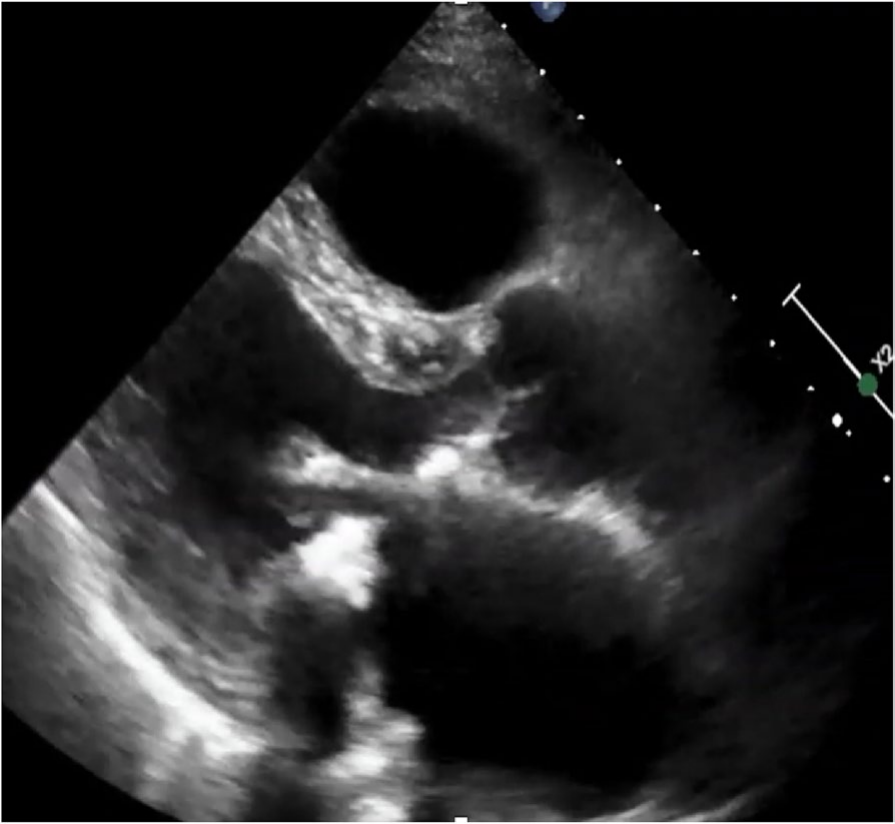
Echocardiography at the initial visit. Transthoracic echocardiography revealed moderate mitral stenosis with bulky mitral annular calcification

Chest radiography revealed mild cardiomegaly. Electrocardiography revealed atrial fibrillation without specific ischemic changes. Computed tomography showed severe MAC (Fig. 1). The calcification extended to a part of the left ventricular myocardium, and there was continuity of calcification between the valve leaflets and the annulus. Transthoracic echocardiography (TTE) revealed moderate mitral stenosis (MS) with bulky MAC (Fig. 2). The cause of heart failure in this case was primarily increased left atrial pressure resulting from mitral stenosis and atrial fibrillation, which led to pulmonary congestion. Additionally, as the patient was receiving dialysis, difficulties in fluid management further contributed to the heart failure. He was symptomatic but was considered high risk for surgery because he was on dialysis with inadequate fluid management and had MAC. First, we decided to manage heart failure and follow-up on an outpatient basis for 2 months, and in case of rapid progression of MS, we intended to perform surgery.

**Fig. 3:**
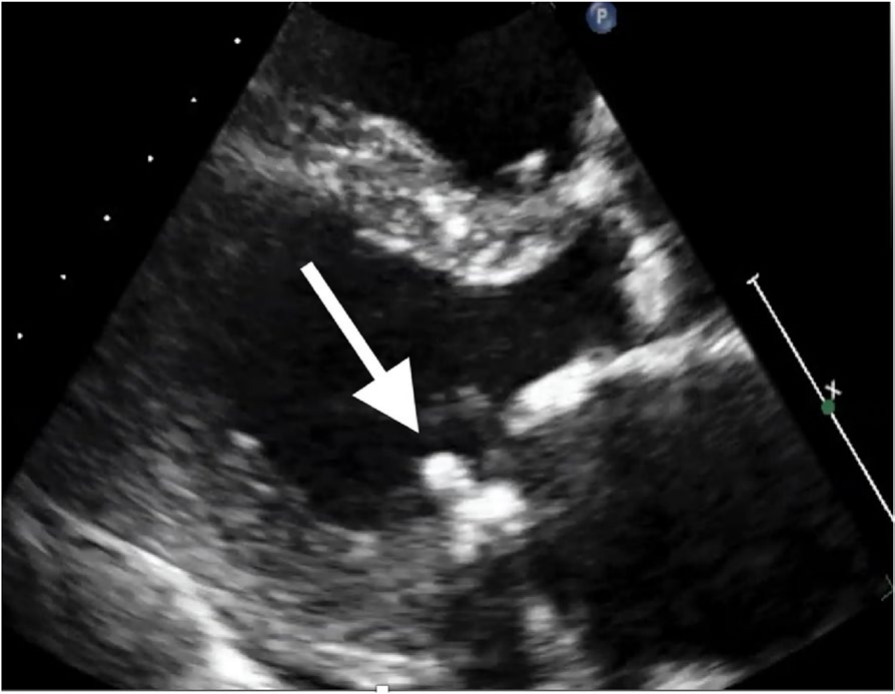
Echocardiography performed 2 months later. Transthoracic echocardiography shows a highly mobile mass (8 × 5 mm) attached to the mitral annulus (white arrow)

Two months later, the patient developed progressive dyspnea, and follow-up TTE revealed a highly mobile mass (8 × 5 mm) attached to the left ventricular (LV) side of the posterior MAC (Fig. 3). No remarkable change was observed in MS progression, and LV systolic function was normal. The mean pressure gradient was 8 mmHg. Left ventricular wall motion was normal, and the ejection fraction was 68.8% (calculated using the Teichholz method). Mitral regurgitation was mild. The reduction in echogenicity within the MAC could not be identified on the preoperative echocardiogram. He was afebrile. Blood investigations revealed hemoglobin 12.2 mg/dL, white blood cells 4750 /μL, platelets 249,000 /μL, and C-reactive protein 0.18 mg/dL. The blood culture results were negative. Preoperative brain MRI and CT scans did not reveal any signs of cerebral infarction. The patient was scheduled for surgery because of congestive heart failure and a high risk of embolization.

**Fig. 4:**
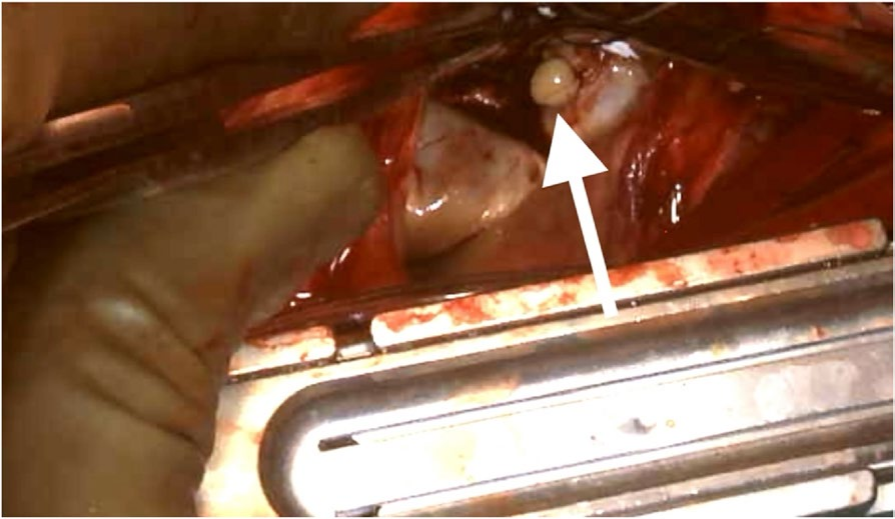
Surgical finding. Toothpaste-like contents drained from the encapsulated mass inside the calcified posterior mitral annulus (white arrow)

**Fig. 5:**
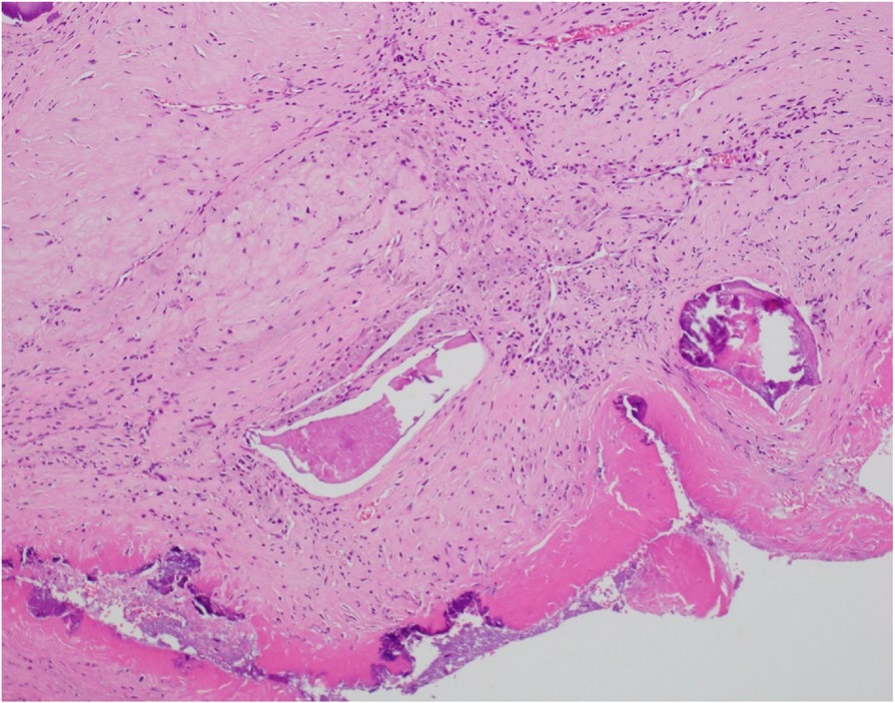
Histopathological finding. Nodular calcifications surrounded by fibrous connective tissue

Surgical inspection revealed that the tumor was attached beneath P3 segment of the mitral valve on the LV side and was removed using forceps without force. When removing the MAC, toothpaste-like contents drained from the encapsulated mass inside the MAC at the P3 segment, where the tumor was located (Fig. 4). The MAC itself was removed using an ultrasonic aspirator, while the caseous calcification of the mitral annulus was paste-like and easily removed with suction and irrigation. The anterior leaflet was partially preserved in the A2 rough zone where there was no calcification and where chordae tendineae were attached. Additionally, a portion of the posterior leaflet was preserved. After reconstructing the defect of the posterior mitral annulus with a bovine pericardial patch to prevent embolization and atrioventricular groove disruption, mitral valve replacement with a mechanical prosthesis, a maze procedure, and left appendage closure were performed. A mechanical valve was chosen based on the consideration of long-term prognosis and concerns about the potential calcification of a bioprosthetic valve due to the patient receiving dialysis, which increases the risk of calcification. Non-everting mattress sutures were used for securing the valve. Histopathological examination revealed that the excised tumor consisted of fibrin and calcium deposits (Fig. 5). Cultures of the excised mitral valve and viscous content tested negative, with no evidence of mycobacteria. The mass was diagnosed as a calcified amorphous tumor (CAT). The patient recovered uneventfully, and was discharged. He was doing well at the 2-year follow-up.

## Discussion and conclusions

Heart CAT is a rare, non-neoplastic cardiac mass first described by Reynolds et al. in 1997 [1]. CAT can be detected in all cardiac chambers, but predominates in the mitral valve or annulus; Surgery can aid in the diagnosis of CAT. CAT may be incidental, and the most common presenting symptoms are dyspnea, embolic events, and syncope. Frequent complications, such as valve disease (31%), MAC (14%), end-stage renal disease (21%), diabetes (14%), and coronary artery disease (12%) have been reported [2]. In the present case, preoperative blood investigations showed no evidence of elevated inflammation and blood cultures were negative; thus, IE was considered negative. However, it is often difficult to distinguish CAT from vegetation or other cardiac tumors using only preoperative data without pathological findings.

The growth rate of CAT is uncertain but it may increase relatively fast, especially MAC-related CAT [2]. The present case showed rapid growth of CAT within a short period of two months. We consider that this rapid progression is norteworthy, particularly in the context of a patient undergoing dialysis with MAC. We speculated that CAT originated from MAC and that MAC may cause further calcification and mass formation, leading to CAT formation. Moreover, MAC may progress to CAT, especially in patients undergoing hemodialysis, owing to its pathology. Abnormalities in calcium-phosphorus metabolism because of renal dysfunction and inflammation associated with hemodialysis may contribute to rapid growth and pathological change [3]. In the Framingham study, the presence of MAC caused a two-fold increase in the risk of stroke after adjusting for multiple risk factors [4]. As MAC itself is a high-risk factor for stroke, MAC-related CAT should be regarded distinct from other CAT.

In conclusion, routine follow-up echocardiography is recommended for patients with MAC, especially those undergoing hemodialysis. It is important to keep in mind that mobile mass lesions with high embolic risk may be observed within a short period of procedure.

## Data Availability

Not applicable.

## Abbreviations

CAT: Calcified amorphous tumor
MAC: Mitral annulus calcification
PCI: Percutaneous coronary intervention
TTE: Transthoracic echocardiography
MS: Mitral stenosis
LV: Left ventricular
